# Adhesin related genes as potential markers for the enteroaggregative *Escherichia coli* category

**DOI:** 10.3389/fcimb.2022.997208

**Published:** 2022-11-08

**Authors:** Claudia A. Freire, Beatriz O. Rodrigues, Waldir P. Elias, Cecilia M. Abe

**Affiliations:** Laboratório de Bacteriologia, Instituto Butantan, São Paulo, Brazil

**Keywords:** enteroaggregative *Escherichia coli*, aggregative-adherence fimbriae, aggregative-forming pilus, AAF, AFP, CS22, adhesins

## Abstract

Enteroaggregative *Escherichia coli* (EAEC) is an important cause of diarrhea in children and adults worldwide. This pathotype is phenotypically characterized by the aggregative-adherence (AA) pattern in HEp-2 cells and genetically associated to the presence of the *aatA* gene. EAEC pathogenesis relies in different virulence factors. At least, three types of adhesins have been specifically associated with EAEC strains: the five variants of the aggregative adherence fimbriae (AAF), the aggregative forming pilus (AFP) and more recently, a fibrilar adhesin named CS22. Our study aimed to evaluate the presence of AAF, AFP and CS22-related genes among 110 EAEC strains collected from feces of children with diarrhea. The presence of *aggR* (EAEC virulence regulator) and genes related to AAFs (*aggA, aafA*, *agg3A*, *agg4A*, *agg5A* and *agg3/4C*), AFP (*afpA1* and *afpR*) and CS22 (*cseA*) was detected by PCR, and the adherence patterns were evaluated on HeLa cells. a*ggR*-positive strains comprised 83.6% of the collection; among them, 80.4% carried at least one AAF-related gene and presented the AA pattern. *aggA* was the most frequent AAF-related gene (28.4% of *aggR*+ strains). *cseA* was detected among *aggR*+ (16.3%) and *aggR*- strains (22.2%); non-adherent strains or strains presenting AA pattern were observed in both groups. *afpR* and *afpA1* were exclusively detected among *aggR*- strains (77.8%), most of which (71.4%) also presented AA pattern. Our results indicate that AAF- and AFP-related genes may contribute to identify EAEC strains, while the presence of *cseA* and its importance as an EAEC virulence factor and genotypic marker needs to be further evaluated.

## Introduction

Enteroaggregative *Escherichia coli* (EAEC) is an important worldwide spread intestinal pathogen that has been related to acute and persistent diarrhea in children and adults from both developed and developing countries, travelers’ diarrhea and outbreaks of diarrhea associated with ingestion of contaminated food and water (Estrada-Garcia and Navarro-Garcia, 2012; Hebbelstrup-Jensen et al., 2014; Gomes et al., 2016). This pathotype was first described by Nataro et al. (1987) after observing that *E. coli* strains isolated from Chilean children with diarrhea presented a stacked-brick-like pattern when adhered on the surface of HEp-2 cells and between them on the coverslip (Nataro et al., 1987) in adherence assays (Cravioto et al., 1979). Since then, this so-called aggregative adherence (AA) pattern has been used as a gold standard to phenotypically define the EAEC category.

A number of EAEC virulence factors, either chromosome- or plasmid-borne, are intimately related to the presence of *aggR*, a transcriptional regulator belonging to the Ara-C family present in the EAEC virulence plasmid called pAA (Morin et al., 2013). Among other important EAEC virulence factors, pAA harbors *aap* and *aatA* genes, which respectively encodes the protein dispersin and its translocator (Sheikh et al., 2002; Nishi et al., 2003). The latest, formerly known as the pCVD432 probe (Baudry et al., 1990), has been widely used to genetically characterize *E. coli* strains belonging to this category (Cerna et al., 2003; Robins-Browne et al., 2004; Sarantuya et al., 2004; Jenkins et al., 2006; Panchalingam et al., 2012; Houpt et al., 2014; Patzi-Vargas et al., 2015; Gomes et al., 2016; Hebbelstrup-Jensen et al., 2017).

Among EAEC virulence factors related to adherence and regulated by AggR, the aggregative adherence fimbriae (AAF), which belongs to the usher-chaperone fimbriae family, have been reported as responsible for the AA pattern. So far, five variants of AAF have been described (AAF/I to V) (Savarino et al., 1994; Czeczulin et al., 1997; Bernier et al., 2002; Boisen et al., 2008; Jønsson et al., 2015). Although there are notable differences between the major pillin-encoding genes of each AAF type, the other genes composing the operon (usher, chaperone and minor pilin) are highly conserved (Savarino et al., 1994; Boisen et al., 2008; Jønsson et al., 2017a). Whilst AAF types seem to occur individually among EAEC strains, the occurrence of strains simultaneously carrying genes for both AAF/III and AAF/V has been also reported (Jønsson et al., 2017b; Dias et al., 2020; Petro et al., 2020).

Recently, Petro et al. (2020) and Boisen et al. (2020) observed the presence of the CS22 related gene *cseA* associated with the presence of *aggR* in EAEC strains. This gene encodes the structural subunit of the fibrilar adhesin CS22, which has been firstly described as a colonization factor in enterotoxigenic *E. coli* (ETEC) (Pichel et al., 2000). Although none of the strains presenting *cseA* (*cseA*+) analyzed by Petro et al. (2020) and Boisen et al. (2020) presented the AA pattern in adherence assays using HEp-2 cells, Boisen et al. (2020) showed that the *cseA*+ strain C671-15 adhered to colonic organoid similarly to the EAEC prototype strain 042.

While analyzing a heteropathogenic enteroaggregative/enterohemorrhagic *E. coli* (EAEC/EHEC) strain showing AA pattern, Lang et al. (2018) identified a new plasmid lacking *aggR* which harbored the *aatA* and *aap* genes. This plasmid (pAFP) also carried a new adhesin named aggregative-forming pilus (AFP) encoded by the *afp* operon, which among its components carries an AraC-like regulator very similar to *aggR*, named *afpR*. AFP was shown to be involved in bacterial piliation, autoaggregation, adhesion and cytotoxicity (Lang et al., 2018) and was recently observed mediating AA in a hybrid enteroaggregative/uropathogenic *E. coli* (EAEC/UPEC) strain (Schüroff et al., 2021; Schüroff et al., 2022). Moreover, phylogenetic analyses have shown that AFP-positive strains carrying other virulence genes related to EAEC pathogenesis (*aat* operon, type 6 secretion system genes and *aap*), are clustered with other fecal EAEC strains (Schüroff et al., 2021).

The present study aims to evaluate the presence of genes related to AAF, CS22 and AFP in a collection of EAEC strains isolated from diarrheic feces and previously classified by the presence of *aatA* gene, in order to identify genetic marker(s) that will allow us to specifically define the EAEC category.

## Materials and methods

### Bacterial strains

A total of 110 *E. coli* strains, characterized as EAEC by the presence of *aatA*, were considered for the present study. These strains were previously obtained from fecal samples collected from children (from 1 to 10 years) with acute diarrhea, in an epidemiological study carried out in the city of Salvador, Brazil (Bueris et al., 2007). Strains were kept at -80˚C in Luria-Bertani (LB) broth containing 15% glycerol and were routinely cultivated on MacConkey or LB agar.

### Adherence assays

Qualitative adherence-assays were performed according to the method described by Cravioto et al. (1979) with modifications. HeLa cells (ATCC CCL-2) were cultured in Dulbecco modified Eagle medium (DMEM) (Cultilab, Brazil) containing 10% fetal bovine serum (FBS), using 24-well plates (Corning, USA) containing a glass coverslip in each well, until reaching 70% confluency (~48 h). Bacterial strains statically grown for 18 h at 37˚C in LB broth were diluted (1:50) in DMEM containing 2% FBS and 1% D-mannose, and inoculated on HeLa cells. After two periods of 3 h of incubation at 37˚C in 5% CO_2_, including a medium change between these periods, each well was washed with phosphate-buffered saline (PBS), fixed with methanol, stained with May-Grunwald and Giemsa (Merck Millipore, USA), and examined by light microscopy.

### DNA isolation

One colony isolated from LB agar culture grown for 18 h at 37°C was selected and mixed with 200 µL of ultra-pure water and incubated at 100˚C for 10 min. After boiling, the lysates were incubated in an ice-bath for 5 min and centrifuged at 1,300 x g for 5 min. The supernatants were collected and stored at -20˚C.

### Gene detection

The detection of the targeted virulence genes was performed by polymerase chain reactions (PCR). Simplex reactions were used to detect the presence of *aggR*, encoding the master EAEC virulence regulator (Morin et al., 2013); *agg5A*, encoding the AAF/V major pilin subunit (Jønsson et al., 2015); *cseA*, encoding the ETEC colonization factor CS22 (Pichel et al., 2000); and *afpA1* and *afpR*, which are part of the AFP-encoding operon (Lang et al., 2018). Multiplex reaction was used to detect the genes encoding the major pilin subunits of AAF/I to IV (*aggA*, *aafA*, *agg3A* and *agg4A*, respectively), and *agg3/4C*, corresponding to the usher of AAF/III, IV and V, (Savarino et al., 1994; Czeczulin et al., 1997; Bernier et al., 2002; Boisen et al., 2008; Jønsson et al., 2015). **Table 1** shows primer sequences and concentrations, amplicon sizes, annealing temperatures and positive controls used in each reaction.

**Table 1:** – Oligonucleotide sequences and PCR conditions used for adhesin-encoding genes detection.

Genes	Oligonucleotides (5´-3´)	Amplicon (bp)	Annealing temperature (˚C)	Positive controls^a^	Reference
*aggR*	(F) CGATACATTAAGACGCCTAAAG	339	56	EAEC 042	Andrade et al. (2014)
	(R) CTGATACATTAAATTCATCTGC				
*aggA*	(F) TCTATCTRGGGGGGCTAACG	218	60	*aggA*: EAEC 17-2 *aafA*: EAEC 042 *agg3A*: EAEC RN785-1 *agg4A* and *agg3/4C*: EAEC BA1116 (GenBank accession number: ON920916)	Jønsson et al. (2017b)
	(R) ACCTGTTCCCCATAACCAGAC				
*aafA*	(F) CTACTTTATTATCAAGTGGAGCCGCTA	292			
	(R) TAGGAGAGGCCAGAGTGWATCC				
*agg3A*	(F) AGCTAGTGCTACTGCAAAATTAAAGTT	359			
	(R) CAGGTTTAATATATTGGTCTGGAATAAC				
*agg4a*	(F) TGAGTTGTGGGGCTAYCTGGA	169			
	(R) CACCATAAGCCGCCAAATAAGC				
*agg3/4C*	(F) CATARTGAAGGTATAACATTTGGTCAGA	477			
	(R) GTCAGCATAACACTTACTGTTCATTC				
	(R) GTAGTTTGCATAGCAATGGCTATTCATT				
*agg5A*	(F) GTTTCATCAACTGGAACTATTACTATTT	401	57	EAEC BA120 (GenBank accession number: ON920918)	Jønsson et al. (2017b)
	(R) TAATTTAAGCTGAAGAATCCAGTCAAT				
*afpA1*	(F) AGAAGCGTAAAAGCTCCCTCC	140	55	UPEC-46	This study
	(R) ACGGTGCTCTGAGTCTTGTT				
*afpR*	(F) GTGAAGAACATTATTGAAGGGGGC	307			Lang et al. (2018)
	(R) CATCACTTAATCGCCAGCGTT				
*cseA*	(F) CGCAAATGCCGCAACTGTA	348	55	EAEC BA249 (GenBank accession number: ON920917)	This study
	(R) GCGTCTGGCAAATTCCAAC				

a Control strains references: EAEC 042 (Elias et al., 1999); EAEC 17-2 (Nataro et al., 1992); EAEC BA120, EAEC BA249 and EAEC 1116 (Bueris et al., 2007); EAEC RN785-1 (Zamboni et al., 2004); UPEC-46 (Schüroff et al., 2021).

PCR was performed in a final volume of 25 µL containing the 20-35 pmol of each primer; dATP, dTTP, dCTP and dGTP (0.1 mM each); 1.5 U Taq DNA polymerase (Invitrogen, USA); 5.0 µL 10X PCR buffer (Invitrogen), MgCl_2_ (1.5-2.0 mM) and 1.0 µL of DNA template. Cycling was conducted as follows: 1 x 94˚C/5 min; 30 x (94˚C/1 min, primer specific annealing temperature presented in **Table 1**/1 min, and 72˚C/1 min), followed by a final cycle of 72˚C/5 min. Amplicons were analyzed by 0.7% (simplex PCR) or 2% (multiplex PCR) agarose gel electrophoresis in Tris-borate-EDTA buffer using the 1 kb ladder (Invitrogen) as marker. The gels were stained with UniSafe Dye (Uniscience) and the amplicons were visualized in a UV transiluminator (Alliance HD 6, Uvitec, UK). *E. coli* HB101 or DH5α were used as negative controls. Positive controls are listed in **Table 1**.

## Results

### The AA-pattern is presented by most of the *aatA*-positive strains

Among the 110 *aatA*+ EAEC strains submitted to adherence assays with HeLa cells, 97 (88.2%) presented the AA pattern. One strain presenting chain-like adherence (CLA) and one presenting localized adherence (AL) patterns were also observed. Eleven strains (10%) were non-adherent (NA) (**Table 2**).

**Table 2:** Adhesin related genes and adherence pattern of 110 *aatA*+ EAEC strains studied.

*aggR/*Adhesin/Gene profile			Adherence pattern				Total
			AA	CLA	LA	NA	
***aggR*+**							
AAF (n=74)	AAF/I	*aggA*	21	–	–	–	21
	AAF/II	*aafA*	14	–	–	–	14
	AAF/III	*agg3A*/*agg3/4C*	12	–	–	–	12
	AAF/IV	*agg4A*/*agg3/4C*	9	–	–	–	9
	AAF/V	*agg5A/agg3/4C*	17	–	–	–	17
	AAF/III-V	*agg3A*/*agg3/4C/agg5A*	1	–	–	–	1
CS22 (n=15)	*cse*A		8	1	–	6	15
AAF-/AFP-/CS22- (n=3)	–		3	–	–	–	3
***aggR-***							
AFP (n=14)	*afpA1*/*afpR*		10	–	1	3	14
CS22 (n=4)	*cseA*		2	–	–	2	4
**Total**			97	1	1	11	110

AA, aggregative adherence; CLA, chain-like adherence; LA, localized adherence; NA, non-adherent.

### Genetic characterization shows an important diversity of EAEC related genes

*aggR* was detected in 92 out of 110 (83.6%) *aatA*+ EAEC strains analyzed. Among the *aggR*+ strains, 74 (80.4%) presented one of the genes encoding the AAF major pilin subunits (**Table 2**). *aggA* was the most frequent among them, detected in 21 of these strains (28.4%), followed by *agg5A* (17 strains/22.9%), *aafA* (14 strains/18.9%), *agg3A* (12 strains/16.2%) and *agg4A* (9 strains/12.2%). Except for one strain (1.4%), harboring *agg3A* and *agg5A*, strains did not harbor more than one gene encoding the major pilin subunit. Finally, *agg3/4C* was detected in all strains harboring *agg3A*, *agg4A* and *agg5A* (39 strains/52.7%). *cseA* was detected in 15 of the 92 *aggR*+ strains (16.3%). The remaining three *aggR*+ strains (3.3%) did not carry either *cseA* or AAF-encoding genes. AFP-encoding genes were not detected among *aggR*+ strains.

Among the 18 *aggR*- strains, 14 (77.8%) carried both *afpR* and *afpA1* genes, while four (22.2%) were *cseA*+. AAF-encoding genes were not detected among *aggR*- strains.

**Figure 1:**
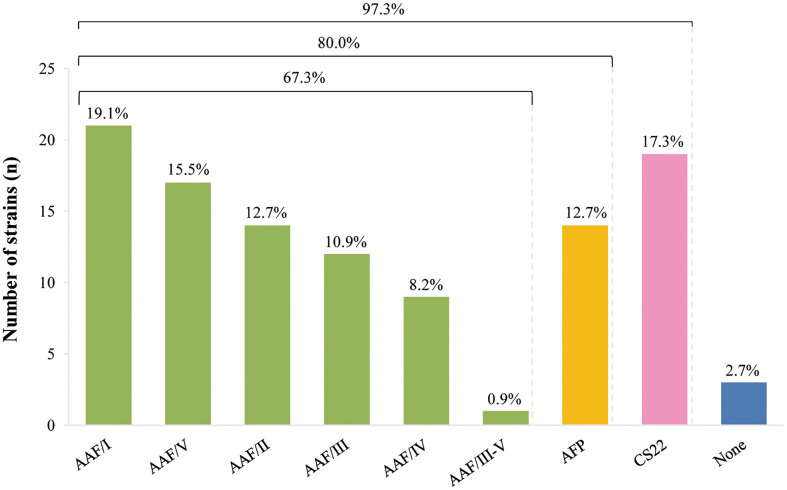
Distribution of AAF, AFP and CS22 related genes among 110 *aatA*+ EAEC strains.

Finally, we emphasize that AAF, AFP and CS22-encoding genes were not simultaneously detected in any of the studied strains. **Figure 1** shows the frequency of strains carrying genes related to AAF (67.3%), AFP (12.7%) and CS22 (17.3%) among the 110 *aatA*+ EAEC strains, independently of the presence of *aggR*. Only 2.7% were devoid of any of these genes.

## Discussion

The 110 *E. coli* strains selected for this study were previously classified as EAEC by the presence of the *aatA* gene, using a multiplex PCR to directly detect strains belonging to four main diarrheagenic *E. coli* categories (enteropathogenic *E. coli*, EPEC; Shiga-toxin producing/enterohaemorrhagic *E. coli*, STEC/EHEC; ETEC and EAEC), during an epidemiological case-control study of the etiology of acute diarrhea (Bueris et al., 2007).

Adherence assays, which correspond to the phenotypic gold standard method used for EAEC characterization, were performed using HeLa cells in order to access the adherence pattern of *aatA*+ EAEC strains, and correlate it with the presence of different adhesins.

Among the 110 strains, 97 (88.2%) strains presented the AA pattern on HeLa cells showing that, in our collection, strains bearing the EAEC characteristic genetic marker *aatA* do not completely correspond to strains presenting AA pattern.

It is well known that *aggR* is present in the majority of EAEC strains (Hebbelstrup-Jensen et al., 2014; Rogawski et al., 2017; Lima et al., 2018; Boisen et al., 2020; Levine et al., 2020; Meza-Segura et al., 2020), and regulates different virulence factors in these strains. Although *aggR* has an important role in EAEC pathogenesis, it is also known that not all *E. coli* strains presenting the AA pattern harbor *aggR*. In fact, the classification of EAEC category in two different groups considering the presence or absence of *aggR* have been proposed: typical EAEC (tEAEC; AA pattern and *aggR*+) and atypical EAEC (aEAEC; AA pattern and *aggR*-) (Kaper et al., 2004; Harrington et al., 2006). According to this definition, our results showed that among 97 strains presenting AA pattern, 85 (87.6%) were tEAEC (*aggR*+), and, 12 (12.4%) were aEAEC (*aggR*-). The presence of *aggR* was also detected in one strain presenting CLA pattern (0.9%), and in other six (5.5%) NA *aatA*+ EAEC strains.

It has been suggested that the presence of *aggR* in tEAEC confers more virulence to these strains (Kaper et al., 2004; Estrada-Garcia and Navarro-Garcia, 2012; Hebbelstrup-Jensen et al., 2014). However, it is important to note that aEAEC strains have also been responsible for important diarrhea outbreaks (Čobeljić et al., 1996; Itoh et al., 1997). Guerrieri et al. (2019) also showed that in a *Galleria mellonella* infection model, aEAEC presented virulence levels comparable to the ones observed to tEAEC. Therefore, AA/*aggR*- (aEAEC) strains should also be considered as an important diarrheagenic agent despite the absence of the *aggR* regulon.

Among EAEC virulence factors regulated by *aggR*, the aggregative adherence fimbriae (AAF) have been associated with the AA pattern in different EAEC prototype strains (Savarino et al., 1994; Czeczulin et al., 1997; Bernier et al., 2002; Boisen et al., 2008; Jønsson et al., 2015). In the present work, genes related to at least one of five AAF types (AAF/I to V) described to date were detected in 74 out of 92 (80.4%) *aggR*+ strains. Among them, the AAF/I major pilin subunit *aggA* (28.4%) was the most frequent pilin-encoding gene found, followed by *agg5A* (22.9%), *aafA* (18.9%), *agg3A* (16.2%) and *agg4A* (12.2%). Although a heterogeneous frequency profile of AAF pilin-encoding genes have been reported among different EAEC strains collections, *aggA* also featured as the most frequent AAF pilin related gene in some studies, ranging from 17.8 - 26.4% (Jønsson et al., 2015; Jønsson et al., 2017b; Boisen et al., 2020; Petro et al., 2020). Other studies respectively pointed to *aafA* (21.8%) (Chattaway et al., 2017), *agg4A* (14.5%) (Hebbelstrup-Jensen et al., 2017), and *agg5A* (20%) (Dias et al., 2020) as the most frequent AAF pilin related genes. It is of notice that all AAF+ strains herein detected also presented the AA pattern on HeLa cells.

One strain harboring genes of the operons simultaneously encoding AAF/III and AAF/V (*agg3A* and *agg5A*, respectively) was also detected in our collection. Similar strains encoding AAF/III- and AAF/V-related genes have been reported in other studies (Jønsson et al., 2017b; Dias et al., 2020; Petro et al., 2020) and although transcription of both *agg3A* and *agg5A* has been shown in some of these strains (Jønsson et al., 2017b), further details on the fimbria structure and its regulation mechanisms remain unknown.

Boisen et al. (2020) recently described the presence of a CS22-like gene cluster among EAEC strains harboring *aggR*. This cluster is an operon highly homologous to the one encoding an ETEC colonization factor named CS22 (Pichel et al., 2000). A frequency of 3.1% among 97 EAEC (*aatA*+ and/or *aaiC*+) strains carrying a complete CS22-like gene cluster was reported by Boisen et al. (2020), while Petro et al. (2020) reported the occurrence of 16.1% among 56 EAEC (*aatA*+ and/or *aaiC*+) strains harboring *cseA*. These strains, however, were respectively minimal or not adherent to HEp-2 cells in adherence assays.

In the present work, 19 (17.3%) *aatA+* EAEC strains harboring the *cseA* marker were detected. However, in contrast to what have been previously reported by Boisen et al. (2020) and Petro et al. (2020), where all the CS22+ strains were also *aggR*+, our study showed that only 15 out of 19 (78.9%) *cseA*+/*aatA*+ EAEC strains were *aggR*+. We also found in HeLa adherence assays that eight (53.3%) of these strains presented the AA pattern and one (6.7%) the CLA pattern; while the remaining six strains (40.0%) were NA. These results show that despite no correlation between presence of *cseA* and adherence pattern was found, some strains presented the AA pattern on HeLa cells. Likewise, we also found two *cseA*+/AA strains among four *aggR*- strains (two presenting the AA pattern and two NA). Although Boisen et al. (2020) reported that three *cseA*+ EAEC were non-adherent on HEp-2 cells, they also observed that one of these strains was able to adhere to human intestinal epithelium in a colonoid model. In this sense, the presence of CS22-encoding genes and their role in EAEC adherence, as well as their regulation by AggR are still unclear. In fact, CS22 is an important virulence factor involved in ETEC colonization (Pichel et al., 2000) and there are no reports concerning the presence of *aggR* (which belongs to the AraC regulator family) in ETEC, suggesting that CS22 related genes are not under exclusive *aggR* regulation.

The AFP biogenesis-related genes share high similarity with the genes composing the operon encoding the bundle-forming pili (BFP), a type IV pilus associated with localized adherence pattern of EPEC strains (Lang et al., 2018). Furthermore, Schüroff et al. (2021) showed that AFP are expressed as thin rigid structures, instead of the characteristic BFP bundles and mediates the AA pattern of a hybrid EAEC/uropathogenic *E. coli* (UPEC) strain isolated from a urinary infection case. In another study, Dias et al. (2020) also reported 15 *aatA*+/*aggR*- strains (6.8%) out of 220 *aatA*+ EAEC strains harboring AFP-related genes; among which, 12 also presented the AA pattern in HeLa cells adherence assays.

Among the 110 *aatA*+ EAEC strains studied, the present work detected 14 strains harboring AFP-encoding genes (*afpA1* and *afpR*); all of them lacking *aggR*. Among these 14 *aggR*-/AFP+/*aatA*+ EAEC strains, 10 (71.4%) presented the AA pattern on HeLa cells, two were NA and one presented LA. As the LA pattern is a phenotypic characteristic of EPEC strains related to the production of BFP (Gomes et al., 2016), the occurrence of this adherence pattern in one aEAEC strain needs to be further studied.

Results obtained in this study draw our attention to the fact that among the 110 *aatA*+ EAEC strains studied, AAF, CS22 or AFP were exclusively present in 107 strains; respectively, 74 (67.3%), 19 (17.3%), and (14) 12.7%. Also important to note that 100% of these 74 AAF+ strains were *aggR*+ and presented AA pattern in HeLa cells adherence assays, while 100% of AFP+ strains were *aggR-*. While the role of AAF in pathogenesis and as a specific EAEC marker is well stablished, recent data showed that AFP has emerged as an important virulence trait in a subset of EAEC strains carrying *aat*, *aai* and *aap* (Lang et al., 2018; Dias et al., 2020; Schüroff et al., 2021; Schüroff et al., 2022). Considering our results, and the EAEC classification proposed by Kaper et al. (2004), we suggest that the search for AAF- and AFP-encoding genes may contribute to genotypically identify and discriminate EAEC strains. Regarding CS22, our results shows that it is not exclusively related to the presence of *aggR*, nor to the AA pattern. As mentioned earlier, CS22 has been described as a virulence factor involved in ETEC colonization (Pichel et al., 2000), but no other significant data on its role in EAEC pethogenesis has been reported. Unlike AAF and AFP, the role and importance of CS22 as a virulence factor in EAEC strains remains virtually unexplored and needs to be validated as an EAEC marker in other EAEC collections.

The present study also showed that the majority of our EAEC strains (80%) harbored either one AAF variant or AFP, highlighting their importance as target antigens to be used in EAEC diagnosis and/or prevention strategies. Further studies including *aatA*- EAEC strains are necessary to evaluate the specificity of theses gene markers.

## Data availability statement

The raw data used as reference for our analyses are available in the Butantan Institute Repository (https://repositorio.butantan.gov.br/handle/butantan/4425).

## Author contributions

WE and CA conceived and supervised the research. CF, BR, and CA conducted the experiments. CF, WE, and CA conducted the analyses, wrote and reviewed the manuscript. All authors contributed to the article and approved the submitted version.

## Funding

This study was supported by grant 2018/04144-0 from São Paulo Research Foundation (FAPESP) to WE.

## Acknowledgments

We would like to thank Dr Paulo Schüroff for designing the primers for *afpA1* genes.

## Conflict of interest

The authors declare that the research was conducted in the absence of any commercial or financial relationships that could be construed as a potential conflict of interest.

## Publisher’s note

All claims expressed in this article are solely those of the authors and do not necessarily represent those of their affiliated organizations, or those of the publisher, the editors and the reviewers. Any product that may be evaluated in this article, or claim that may be made by its manufacturer, is not guaranteed or endorsed by the publisher.
